# Two Trichothecene Mycotoxins from *Myrothecium* *roridum* Induce Apoptosis of HepG-2 Cells via Caspase Activation and Disruption of Mitochondrial Membrane Potential

**DOI:** 10.3390/molecules21060781

**Published:** 2016-06-17

**Authors:** Wei Ye, Yuchan Chen, Haohua Li, Weimin Zhang, Hongxin Liu, Zhanghua Sun, Taomei Liu, Saini Li

**Affiliations:** State Key Laboratory of Applied Microbiology Southern China, Guangdong Provincial Key Laboratory of Microbial Culture Collection and Application, Guangdong Open Laboratory of Applied Microbiology, Guangdong Institute of Microbiology, Guangzhou 510070, China; yewei@gdim.cn (W.Y.); chenyc@gdim.cn (Y.C.); Lihh@gdim.cn (H.L.); hxinlui1225@163.com (H.L.); sunzh@gdim.cn (Z.S.); ltm840801@163.com (T.L.); lisn@gdim.cn (S.L.)

**Keywords:** epiroridin acid, mytoxin B, apoptosis, caspase cascade, mitochondrial membrane potential

## Abstract

Trichothecene mycotoxins are a type of sesquiterpenoid produced by various kinds of plantpathogenic fungi. In this study, two trichothecene toxins, namely, a novel cytotoxic epiroridin acid and a known trichothecene, mytoxin B, were isolated from the endophytic fungus *Myrothecium roridum* derived from the medicinal plant *Pogostemon cablin*. The two trichothecene mytoxins were confirmed to induce the apoptosis of HepG-2 cells by cytomorphology inspection, DNA fragmentation detection, and flow cytometry assay. The cytotoxic mechanisms of the two mycotoxins were investigated by quantitative real time polymerase chain reaction, western blot, and detection of mitochondrial membrane potential. The results showed that the two trichothecene mycotoxins induced the apoptosis of cancer cell HepG-2 via activation of caspase-9 and caspase-3, up-regulation of *bax* gene expression, down-regulation of *bcl-2* gene expression, and disruption of the mitochondrial membrane potential of the HepG-2 cell. This study is the first to report on the cytotoxic mechanism of trichothecene mycotoxins from *M. roridum*. This study provides new clues for the development of attenuated trichothecene toxins in future treatment of liver cancer.

## 1. Introduction

Primary liver cancer is a common malignant tumor, and about 260,000 people die from liver cancer all over the world every year. The five-year survival rate of liver cancer patients after surgery remains below 40%. Thus, effective therapeutic agents for the treatment of liver cancer must be developed. Chlorotoxin, extracted from scorpions, was reported to be used for the treatment of neuroglioma with evident clinical efficacy and low side-effects [1]. Botulinum toxin A was also reported to treat overactive bladder with high efficiency [2], which demonstrated the great potential of toxins in cancer treatment combined with targeting factors such as EGFR and single-chain variable fragments (scFvs) [3,4]. MT-2H74 is an engineered toxin body comprised of scFv and a modified ribosome-inactivating protein derived from Shiga-like toxin 1 A. MT-2H74 demonstrated effective cell killing at picomolar concentrations on cell lines expressing high levels of HER2, showing great therapeutic efficacy against breast carcinomas [5]. rGel/EGF is a type of chimeric toxin, which is composed of the recombinant plant toxin gelonin and epidermal growth factor receptor (EGFR). The photochemical internalization of rGel/EGF was shown to be highly effective against EGFR-expressing cell lines, including head and neck squamous cell carcinoma cell lines [6]. Attenuated diphtheria toxin A has great potential to be used as a drug for cancer treatment with the advantage of lower side-effect [7]. Therefore, different types of attenuated toxins with high efficacy should be developed against tumor cell lines and reduced toxicity toward human normal cells, thus promoting the application of toxins in cancer therapy.

Trichothecene mycotoxins are a family of chemically-related mycotoxins produced by various species of *Fusarium*, *Trichoderma*, *Trichthecium*, *Cephalosporium*, *Myrothecium*, *Verticillium*, and *Stachybotrys* [8]. The trichothecene mycotoxins are the main pathogenic factors of pathogenic fungus *Fusarium graminearum*, the virulence of which will be attenuated dramatically if this fungus could not produce trichothecene mycotoxins [9]. Trichothecene mycotoxins can inhibit and disturb the synthesis of DNA and proteins. T-2 and Don toxins are famous trichothecene mycotoxins produced by *F. graminearum*. T-2 toxin could cause the damage of cell membrane and induce the apoptosis of human lymph nodes and hematopoietic cells [10].

Trichothecenes were found to display significant cytotoxic activity against tumor cell lines. Trichothecene mycotoxins, including T-2 and DON toxins, were reported to show strong cytotoxicities against HepG-2 cells with IC_50_ values lower than 0.1 μM [11,12]. In our previous study, a novel trichothecene, *i.e.*, epiroridin acid, together with two known trichothecene toxins, *i.e.*, mytoxin B and epiroridin E, were isolated from the fermentation liquid of *Myrothecium roridum* derived from the medicinal plant *Pogostemon cablin* [13]. Epiroridin acid, mytoxin B, and epiroridin E showed strong cytotoxicities toward HepG-2 tumor cell line with IC_50_ values of 0.38, 0.005, and 0.004 μM, respectively. Therefore, trichothecene mycotoxins have shown great potential in cancer treatment [12]. However, the cytotoxic mechanism of trichothecene mycotoxins against tumor cells remains unclear. In this study, the cytotoxic mechanisms of mytoxin B and the novel epiroridin acid against HepG-2 tumor cells were investigated by quantitative real time polymerase chain reaction (qRT-PCR), Western blot, and flow cytometry assay. The investigation on the apoptosis mechanism of HepG-2 cells induced by epiroridin acid and mytoxin B would shed light on the utilization of trichotecene toxins in future treatment of liver cancer combined with targeting technology.

## 2. Results

### 2.1. Cytotoxicity Detection of Epiroridin Acid and Mytoxin B

**Figure 1:**
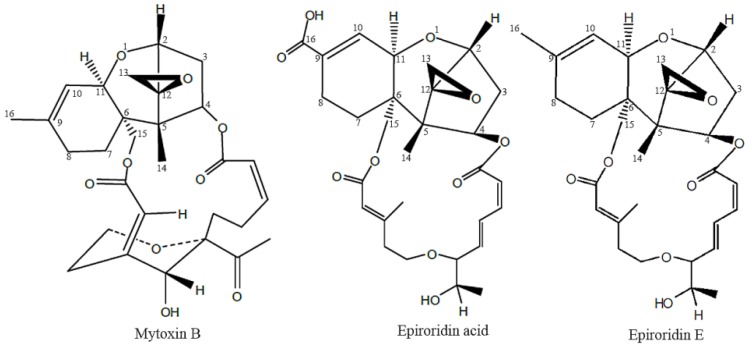
The structures of mytoxin B, epiroridin acid and epiroridin E.

The structures of epiroridin acid and mytoxin B, together with another known trichothecene epiroridin E are shown in Figure 1 [13].

**Figure 2:**
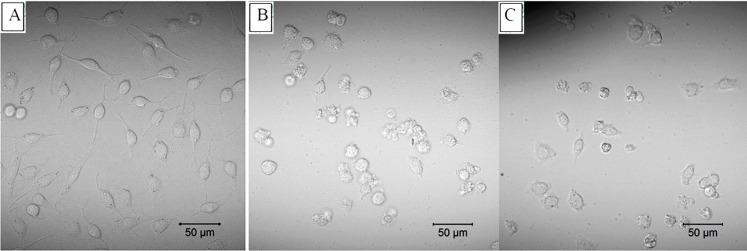
HepG-2 cells were treated with mytoxin B and epiroridin acid for 24 h, respectively, and observed by a laser confocal microscopy: (**A**) untreated HepG-2 cells; (**B**) HepG-2 cells treated with epiroridin acid; (**C**) HepG-2 cells treated with mytoxin B.

**Figure 3:**
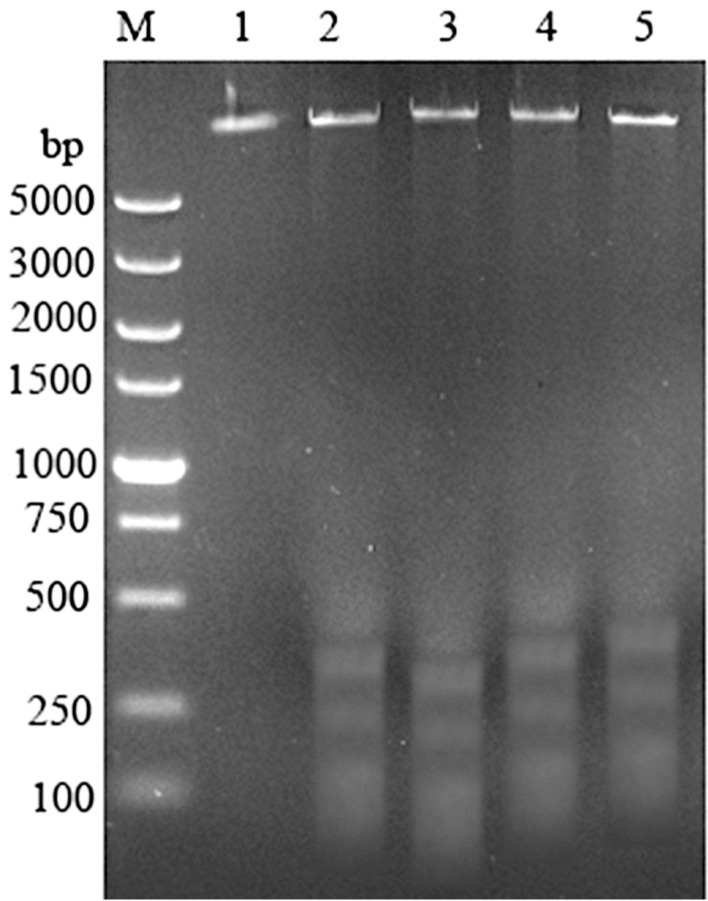
DNA fragmentation of HepG-2 cells treated with mytoxin B and epiroridin acid: M. DNA ladder; (**1**) control; (**2**) HepG-2 cells treated with epiroridin acid for 48 h; (**3**) HepG-2 cells treated with mytoxin B for 48 h; (**4**) HepG-2 cells treated with mytoxin B for 24 h; (**5**) HepG-2 cells treated with epiroridin acid for 24 h.

The cytotoxicity assay results showed that epiroridin acid, mytoxin B, and epiroridin E exhibited significant cytotoxic activities against HepG-2 tumor cells [13]. And the IC_50_ values of mytoxin B and epiroridin acid towards human hepatic stellate cell line LX-2 were 0.004 ± 0.0003 μM, 0.477 ± 0.056 μM, respectively. Mytoxin B showed the same cytotoxicity towards LX-2 and HepG-2, whereas epiroridin acid showed weaker cytotoxicity towards LX-2 cells than that towards HepG-2 cells. Round cells and apoptosis bodies were observed with laser confocal microscopy in epiroridin acid- and mytoxin B-treated HepG-2 cells, and more round and shrinking cells were observed in mytoxin B-treated HepG-2 cells (Figure 2C) than in epiroridin acid-treated cells, whereas no such phenomenon was observed in the control (Figure 2A). DNA fragments were also detected by agarose gel electrophoresis in mytoxin B- and epiroridin acid-treated HepG-2 cells, and slightly more DNA fragments were detected in HepG-2 cells treated with mytoxin B for 48 h than those in epiroridin acid-treated cells, whereas no such DNA fragments were detected in the control (Figure 3).

### 2.2. qRT-PCR Analysis of Mytoxin B- and Epiroridin Acid-treated HepG-2 Cells

**Figure 4:**
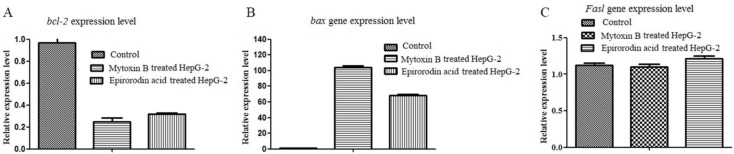
qRT-PCR analysis of the expression levels of *bcl-2*, *bax*, and *Fasl* in HepG-2 cells treated with mytoxin B and epiroridin acid for 24 h, respectively: (**A**) the expression level of *bcl-2* gene; (**B**) the expression level of *bax* gene; (**C**) the expression level of *Fasl* gene.

The total RNAs of mytoxin B- and epiroridin acid-treated cells were extracted, and the expression levels of genes related to the apoptosis of HepG-2 cells were investigated. As shown in Figure 4, the relative expression level of *bcl-2* gene compared with β-actin in control was 0.97 ± 0.05 fold, and the relative expression levels in mytoxin B- and epiroridin acid-treated HepG-2 cells were 0.30 ± 0.02 fold and 0.42 ± 0.01 fold, respectively. This finding demonstrates that treatment with epiroridin acid could down-regulate the expression level of *bcl-2* gene in HepG-2 cells. The relative expression level of *bax* gene in the control was about 1.09 ± 0.04 fold, the relative expression levels of *bax* gene in mytoxin B- and epiroridin acid-treated HepG-2 cells were 104.0 ± 2.5 fold and 68.2 ± 2.09 fold, respectively (Figure 4). The results showed that the expression level of *bax* gene was significantly up-regulated by the treatment with mytoxin B and epiroridin acid. The results shown in Figure 4C manifested that no significant difference exists between the expression level of *Fasl* gene in the control and in HepG-2 cells treated with mytoxin B and epiroridin acid, which was in accordance with the feature of apoptosis. The above results demonstrated that the treatment with mytoxin B and epiroridin acid induced the apoptosis of HepG-2 cells by the up-regulation of *bax* gene and the down-regulation of *bcl-2* gene.

### 2.3. Western Blot Analysis of Mytoxin B- and Epiroridin Acid-treated HepG-2 Cells

**Figure 5:**
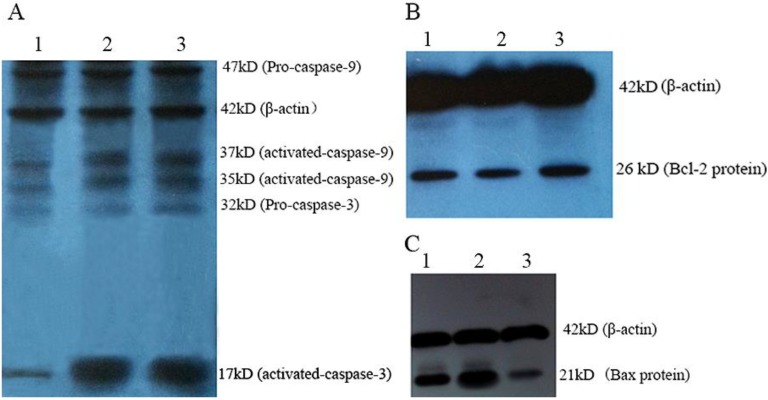
Western blot analysis of the activation of caspases and the expression of Bax and Bcl-2 proteins in HepG-2 cells treated with mytoxin B and epiroridin acid for 24 h, respectively: (**A**) detection of caspase-3 and caspase-9: (**1**) control; (**2**) mytoxin B-treated HepG-2 cells; (**3**) epiroridin acid-treated HepG-2 cells; (**B**) detection of Bcl-2 protein: (**1**) epiroridin acid-treated HepG-2 cells; (**2**) mytoxin B-treated HepG-2 cells; (**3**) control; (**C**) detection of Bax protein: (**1**) epiroridin acid-treated HepG-2 cells; (**2**) mytoxin B-treated HepG-2 cells; (**3**) control.

The expression levels of proteins related to apoptosis were investigated by western blot analysis. The bands corresponding to pro-caspase-3 and pro-caspase-9 were detected in untreated cells and treated cells with nearly the same intensity, and strong bands corresponding to activated caspase-9 (37 and 35 kD) and activated caspase-3 (17 kD) were detected in HepG-2 cells treated with mytoxin B and epiroridin acid, whereas only very weak corresponding bands were detected in untreated HepG-2 cells (Figure 5A).

The relative intensity of Bcl-2 protein band in the control was 2.18 ± 0.16 fold and 1.39 ± 0.21 fold, respectively, as that in the HepG-2 cells treated with mytoxin B and epiroridin acid (Figure 5B). Meanwhile, the relative intensities of Bax protein bands in mytoxin B- and epiroridin acid-treated cells were 4.51 ± 0.29 fold, 2.67 ± 0.18 fold, respectively, as that in the control (Figure 5C), which was analyzed by gel analysis software Bandscan 5.0 (BioMarin Pharmaceutical Inc., Novato, CA, USA), and protein levels were normalized against those of β-actin respectively, data were expressed as mean ± SD (*n* = 3). The western blot analysis results proved that trichotecene mycotoxins mytoxin B and epiroridin acid induced the apoptosis of HepG-2 cells by the activation of caspase-3 and caspase-9 protein as well as by the down-regulation of Bcl-2 protein expression and up-regulation of Bax protein.

### 2.4. Flow Cytometry Assay of HepG-2 Cells Treated With Epororidin Acid and Mytoxin B

**Figure 6:**
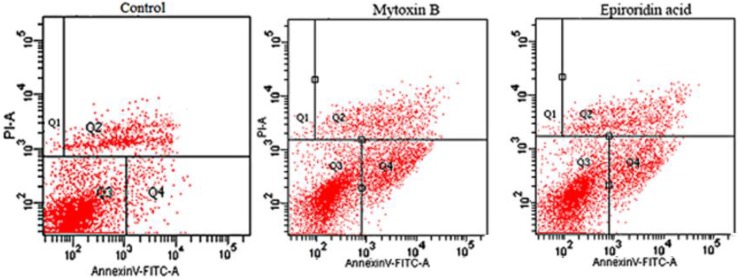
Flow cytometry assay of apoptosis rate of HepG-2 cells treated with mytoxin B and epiroridin acid for 24 h, respectively.

The untreated cells and trichothecene mycotoxins-treated HepG-2 cells were stained by Annex-V and propidium iodide (PI) and loaded onto flow cytometry. It was shown that most cells were in the Q3 region in the control, whereas much less cells were detected in the Q3 region in HepG-2 cells treated with epororidin acid and mytoxin B, indicating the cytotoxicities of these two compounds towards HepG-2 cells (Figure 6). The early apoptosis rates of HepG-2 cells treated with mytoxin B and epiroridin acid were 11.7% and 17.3%, respectively. The terminal apoptosis rates of HepG-2 cells treated with mytoxin B and epiroridin acid were 22.3% and 11.7%, respectively (Figure 6). Untreated HepG-2 cells showed early apoptosis rate and terminal apoptosis rate of 3.4% and 5%, respectively, which was probably due to the natural cellular metabolism. The results showed the higher terminal apoptosis rate, whereas lower early apoptosis rate of mytoxin B-treated cells compared with those of epiroridin acid-treated cells, which was in accordance with the stronger cytotoxicity of mytoxin B than epiroridin acid against HepG-2 cells.

### 2.5. Detection of Mitochondrial Transmembrane Potential

**Figure 7:**
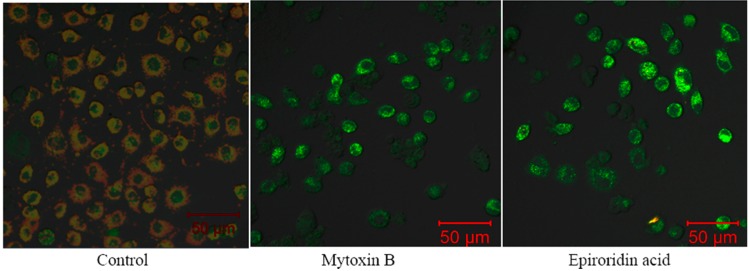
The control and HepG-2 cells treated with mytoxin B and epiroridin acid, which were stained with JC-10 and observed by a laser confocal microscopy.

**Figure 8:**
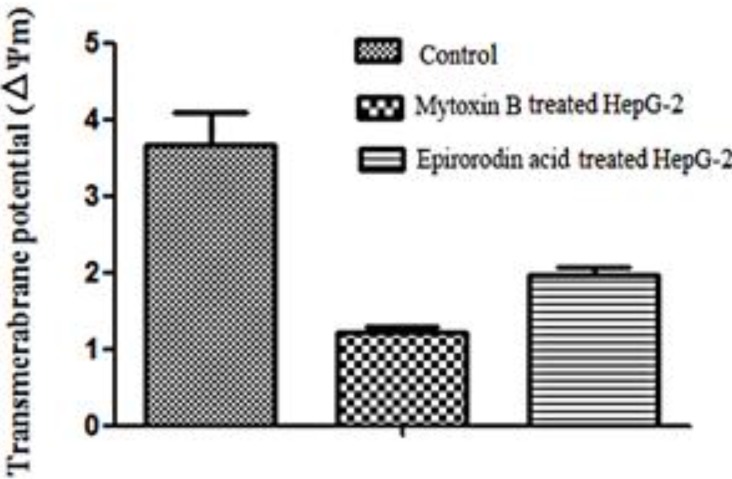
Detection of mitochondrial transmembrane potential of HepG-2 cells treated with mytoxin B and epiroridin acid for 24 h, respectively. The mitochondrial transmembrane potential was calculated as the ratio of red/green fluorescence intensity.

The mitochondrial transmembrane potentials of trichotecene mycotoxins-treated cells were analyzed by the comparison of intensities of green fluorescence and red fluorescence. Very low intensity of green fluorescence intensity and high intensity of red fluorescence were observed by laser confocal microscopy in untreated HepG-2 cells, whereas green fluorescence was observed with much stronger intensity in mytoxin B- and epiroridin acid-treated HepG-2 cells (Figure 7), indicating much lower mitochondrial transmembrane potential. The mitochondrial transmembrane potentials in mytoxin B- and epiroridin acid-treated HepG-2 cells were 1.23 ± 0.09 and 1.97 ± 0.13, respectively, whereas the mitochondrial transmembrane potential of untreated HepG-2 cells was 3.67 ± 0.61 (Figure 8). Meanwhile, the stronger intensity of green fluorescence, the lower mitochondrial transmembrane potential and more round cells were observed in mytoxin B- treated HepG-2 cells compared with epiroridin acid-treated HepG-2 cells, indicating the stronger cytotoxicity of mytoxin B than epiroridin acid (Figure 7). The results demonstrated that the treatment of trichothecene mycotoxins mytoxin B and epiroridin acid could decrease the mitochondrial transmembrane potential of HepG-2 cells, thus inducing the apoptosis of HepG-2 cells.

## 3. Discussion

In this study, cell morphology inspection and DNA fragmentation detection combined with the flow cytometry assay results demonstrated the apoptosis of HepG-2 cells treated by the two trichothecene toxins, mytoxin B and epiroridin acid, isolated from *M. roridum*. The apoptosis mechanisms of which were investigated by qRT-PCR, western blot analysis, and flow cytometry assay. Three trichothecene mytoxins, mytoxin B, epiroridin acid and epiroridin E were isolated from *M.* *roridum* [13]. Mytoxin B and epiroridin E showed nearly the same cytotoxicity because they have the same mother nucleus; meanwhile, mytoxin B and epiroridin acid shared more structure differences in the side chain. Thus, the novel trichothecene epiroridin acid and mytoxin B were selected to compare their apoptosis-inducing effects towards HepG-2 cells and the relationship of their structure differences, and their cytotoxic mechanisms towards HepG-2 cells were also investigated. The weaker cytotoxicity of epiroridin acid towards human normal cells (LX-2) than that towards human tumor cells (HepG-2) suggested the potential application of epiroridin acid in future treatment of liver cancer. To the best of our knowledge, this is the first report on the apoptosis mechanism of the novel trichothecene mycotoxin epiroridin acid and the first study to compare the apoptosis status of epiroridin acid and a known trichothecene mytoxin B systematically.

Epiroridin acid and mytoxin B isolated from *M. roridum* shared the same mother nucleus of 16 member ring with epoxy structure [13]. The carboxyl group was found at C-9 of epiroridin acid, whereas no carboxyl group was found at C-9 of mytoxin B [13]. The presence of carboxyl group at C-9 would increase the acidity of epiroridin acid; meanwhile, this carboxyl group is near the epoxy structure in the mother nucleus, which is critical for the toxicity of trichothecene [12,13,14]. The higher concentration (1.0 μM) of epiroridin acid than mytoxin B (0.1 μM) showed weaker apoptosis effect towards HepG-2 cells, further demonstrating the much weaker cytotoxicity of epiroridin acid than mytoxin B towards HepG-2 cells. The structure differences among epiroridin acid, epiroridin E and mytoxin B as well as the cytotoxicities of the three trichothecene mycotoxins towards HepG-2 cells [13] indicated that the oxygen atom positions in the side chain at C-15 had no great effect on the cytotoxicity. The carboxyl group at C-9 in the mother nucleus of epiroridin acid resulted in the much weaker cytotoxicity of epiroridin acid than that of mytoxin B. It was reported that the modification at C-9 of trichothecene T-2 toxin could reduce the cytotoxicity against mouse lymphoma cells [14]. Lower expression level of *bcl-2* gene and higher expression level of *bax* gene as well as the higher apoptosis rate and lower mitochondrial transmembrane potential were observed in mytoxin B-treated cells compared with epiroridin acid-treated HepG-2 cells. These results further confirmed the stronger cytotoxicity and more significant apoptosis effect caused by mytoxin B compared with that caused by epiroridin acid. The possible reason was that carboxyl group in the side chain of epiroridin acid weakened the cytotoxicity, resulting in the weaker apoptosis effect of epiroridin acid against HepG-2 cells. The relatively lower cytotoxicity of this novel trichothecene mycotoxin epiroridin acid than the known trichothecene would provide clues for the future modification of trichothecene mycotoxin with the purpose of obtaining attenuated toxins with higher specificity and lower side-effect, thereby laying a foundation for future cancer therapy by the utilization of novel toxins [15,16].

Cysteinyl aspartate specific proteinases (caspases) play very important roles during apoptosis [17]. In this study, the mytoxin B and epiroridin acid induced the cleavage of caspase-9 protein to form 37 and 35 kD fragments. The cleaved caspase-9 activated pro-caspase-3 and 17 kD fragment were detected in trichothecene mytoxin-treated HepG-2 cells. The cleaved caspase-3 could hydrolyze target protein, thus leading to the apoptosis of HepG-2 cells. Weak bands of cleaved caspase-3 and caspase-9 were also detected in the control, which could be attributed to the very low apoptosis rate in HepG-2 cells without treatment with mytoxin B and epiroridin acid because of natural cellular metabolism. Bcl-2 and Bax proteins also play important roles in the apoptosis process [17]. We postulated that the decreased expression of Bcl-2 protein in mytoxin B- and epiroridin acid-treated HepG-2 cells could promote the release of cytochrome c, thus promoting apoptosis [17,18]. Bcl-2 protein is a kind of anti-apoptosis protein, which played an important role in the apoptosis of different cells. It was reported that Bcl-2 not only functions as an anti-apoptotic protein, but also as an anti-autophagy protein via its inhibitory interaction with Beclin 1. This anti-autophagy function of Bcl-2 may help maintain autophagy at levels that are compatible with cell survival [19]. Bax protein in mytoxin B- and epiroridin acid-treated HepG-2 cells with much higher expression level compared with control could insert into the mitochondrial outer membrane, thus altering mitochondrial membrane permeability, reducing the mitochondrial membrane potential, and finally enhancing the apoptosis rate significantly. The crude extract of Pien Tze Huang was also reported to induce the apoptosis of human osteosarcoma MG63 cells by the activation of caspase cascades and the alteration of apoptotic mediators *bcl-2* and *bax* expression, further demonstrating the important roles of Bcl-2, Bax protein, and caspase cascades in the apoptosis of tumor cells [20].

## 4. Materials and Methods

### 4.1. Materials

*M. roridum* was isolated from the medicinal plant *P. cablin*. Trichothecene toxins mytoxin B and epiroridin acid were isolated from the fermentation liquid of *M. roridum* in our lab [13]. HepG-2 and LX-2 cell lines were purchased from ATCC (Manassas, VA, USA). The fluorochrome JC-10, PI, and Annexin-V were obtained from Sigma (St. Louis, MO, USA). The qRT-PCR mix was provided by Fermentas (Harrington, WA, USA). The anti-caspase-3 mouse monoclonal antibody, anti-caspase-9 rabbit monoclonal antibodies and rabbit anti-mouse IgG secondary antibody were purchased from CST (Danvers, MA, USA).

### 4.2. Cytotoxicity Detection of Epiroridin Acid and Mytoxin B

HepG-2 and LX-2 cells were adjusted to 3 × 10^4^/mL and seeded onto a 96-well microtiter plate. Different concentrations of mytoxin B and epiroridin acid were dissolved in DMSO and added after 24 h of incubation at 37 °C, 5% CO_2_. Adriamycin was employed as a positive control. After further incubation at 37 °C for 24 h, the inhibitory effect of the added samples on the proliferation of HepG-2 cells was determined by the sulforhodamine B (SRB) method [21]. The control group and trichothecene toxin-treated HepG-2 cells were observed by a laser confocal microscopy (LeicaAF6000, Solms, Germany). The total DNAs of the control group and trichothecene toxin-treated HepG-2 cells were extracted by DNA ladder extraction kit (Beyotime, Shanghai, China) and detected by 1.5% agarose gel electrophoresis.

### 4.3. qRT-PCR Analysis of Genes Related to the Apoptosis

HepG-2 cells were treated with 1.0 μM epiroridin acid and 0.1 μM mytoxin B for 24 h. The cells were collected and washed with phosphate sodium buffer. The total RNAs of untreated HepG-2 cells and trichothecene toxin-treated cells were extracted using a RNA extraction kit (Umagen, Guangzhou, China). The total RNAs were adjusted to the same concentration and used as templates to obtain corresponding cDNAs using the same MasterMix (ABM, Peterborough, ON, Canada). The primes (Table 1) and cDNAs were added to the qPCR Mix (Fermentas) to identify the relative expression levels of genes *bcl-2*, *bax*, and *Fasl*. β-actin was used as a reference gene. qRT-PCR was performed using the Mastercycler ep realplex System (Eppendorf, Hamburg, Germany), and the qRT-PCR thermal cycling condition for all reactions was 95 °C for 1 min 50 s, followed by 40 cycles of 95 °C for 10 s, and 55 °C for 33 s. All reactions were conducted in biological triplicates, and the results were expressed as relative expression levels to β-actin gene. The C_T_ values obtained were used as the original data to calculate the relative expression levels of different genes to histone gene by the 2^−ΔΔ*C*^^T^ method [22].

### 4.4. Western Blot Analysis of Caspase-3, Caspase-9 Protein

HepG-2 cells were treated with 1.0 μM epiroridin acid and 0.1 μM mytoxin B for 24 h. The cells were collected; 500 μL protein extracting buffer (Pierce, Waltham, MA, USA) was added to the cells, and total proteins were extracted and adjusted to the concentration of 1.0 mg/mL. The proteins were loaded to 12% SDS-PAGE with a volume of 10 μL and then transferred onto nitrocellulose membrane at a voltage of 100 V for 45 min. The membrane was blocked by 5% non-fat milk for 3 h and then incubated at 4 °C overnight in anti-caspase-3 and anti-caspase-9 mouse monoclonal antibody with a dilution of 1:2000. After being washed with TBST buffer containing tween-20 for three times, the membrane was incubated in rabbit anti-mouse IgG secondary antibody at 37 °C for 1 h. The target band was finally visualized by ECL kit (Thermo Fisher, Waltham, MA, USA) according to the manufacturer’s instructions.

### 4.5. Flow Cytometry Assay of Trichothecene Mycotoxin-treated HepG-2 Cells

HepG-2 cells with a concentration of 5 × 10^4^/mL were incubated at 37 °C for 24 h. Afterward, 1.0 μM epiroridin acid and 0.1 μM mytoxin B was added to HepG-2 cells. After 24 h of incubation, the cells were collected by centrifugation and then washed with PBS. About 300 μL of binding buffer was used to resuspend cells, and 0.4 μL of Annexin V-FITC with a concentration of 0.3 mg/mL was added and incubated at room temperature away from light for 15 min. Consequently, 2 μL of PI dye with a concentration of 50 μg/mL was added and incubated at room temperature away from light for 5 min. Finally, the stained cells were loaded onto flow cytometry (FC500, Beckman, Brea, CA, USA). The apoptosis of epiroridin acid- and mytoxin B-treated HepG-2 cells were analyzed.

### 4.6. Detection of Mitochondrial Transmembrane Potential

Epiroridin acid (1.0 μM) and mytoxin B (0.1 μM) were added to the HepG-2 cells at the logarithmic phase. After 24 h of incubation, the cells were collected and resuspended in 200 μL PBS. Approximately 0.5 μL of lipophilic cation 5,5′,6,6′-tetrachloro-1,1′,3,3′-tetraethylbenzimidazolcarbocyanine iodide (JC-10) dyed with a concentration of 10 μg/mL was added to the treated HepG-2 cells and incubated at 37 °C for 20 min away from light. The fluorescene of control and treated HepG-2 cells was observed by a laser confocal microscopy. Finally, the stained HepG-2 cells were loaded onto flow cytometry. The mitochondrial transmembrane potential was analyzed by detecting the intensity of red and green fluorescence, and the ratio of red/green fluorescence was calculated.

## 5. Conclusions

In this study, the cytotoxicity mechanisms of a novel trichothecene mycotoxin, epiroridin acid, and a known trichothecene, mytoxin B, from *M.* *roridum* were investigated. The laser confocal microscopy observation results, DNA fragmentation detection and flow cytometry assay results demonstrated the apoptosis of HepG-2 cells treated with epiroridin acid and mytoxin B. The results of qRT-PCR, western blot analysis and flow cytometry assay showed that mytoxin B and epiroridin acid induced the apoptosis of HepG-2 cells by the activation of caspase cascades, the up-regulation of Bax protein and the down-regulation of Bcl-2 protein, thereby reducing the mitochondrial membrane potential. Epiroridin acid-treated HepG-2 cells showed lower apoptosis rate, lower expression level of *bax* gene, higher expression level of *bcl-2* gene as well as higher mitochondrial membrane potential compared with mytoxin B-treated HepG-2 cells. The relatively lower cytotoxicity of epiroridin acid against HepG-2 cells compared with that of mytoxin B, combined with the lower cytotoxicity of epiroridin acid towards LX-2 cells than that of mytoxin B provides a new clue for the development of attenuated trichothecenes toxins, thus shedding light on the future therapy of liver cancer using attenuated toxin with lower side-effect combined with targeting technique.

## Figures and Tables

**Table 1:** Primers for the amplification of genes related to the apoptosis.

Primers	Sequence (5’-3’)
*bcl*-2 F	TTGGCCCCCGTTGCTT
*bcl*-2 R	CGGTTATCGTACCCCGTTCTC
*bax* F	TCCCCCCCGAGAGGTCTTTT
*bax* R	CGGCCCCAGTTGAAGTTG
*Fasl* F	ATCCCTCTGGAATGGGAAGA
*Fasl R*	CCATATCTGTCCAGTACTGC
β-actin F	GGCATCGTAGTGAGCTCCG
β-actin R	GCTGGAAGTGAACGCAG
